# Standardized Effect Sizes for Moderated Conditional Fixed Effects with Continuous Moderator Variables

**DOI:** 10.3389/fpsyg.2017.00562

**Published:** 2017-04-21

**Authors:** Todd E. Bodner

**Affiliations:** Department of Psychology, Portland State UniversityPortland, OR, USA

**Keywords:** statistical interactions, graphs, standardized effect sizes, standardized mean differences, semi-partial correlations

## Abstract

Wilkinson and Task Force on Statistical Inference (1999) recommended that researchers include information on the practical magnitude of effects (e.g., using standardized effect sizes) to distinguish between the statistical and practical significance of research results. To date, however, researchers have not widely incorporated this recommendation into the interpretation and communication of the conditional effects and differences in conditional effects underlying statistical interactions involving a continuous moderator variable where at least one of the involved variables has an arbitrary metric. This article presents a descriptive approach to investigate two-way statistical interactions involving continuous moderator variables where the conditional effects underlying these interactions are expressed in standardized effect size metrics (i.e., standardized mean differences and semi-partial correlations). This approach permits researchers to evaluate and communicate the practical magnitude of particular conditional effects and differences in conditional effects using conventional and proposed guidelines, respectively, for the standardized effect size and therefore provides the researcher important supplementary information lacking under current approaches. The utility of this approach is demonstrated with two real data examples and important assumptions underlying the standardization process are highlighted.

A statistical two-way interaction implies that the size and perhaps the direction of a focal relationship between two variables depends on the value of a third variable, the moderator variable. The numerous examples of statistical interactions that appear in basic and applied research reports clearly indicate the importance of interactive effects in the social and behavioral sciences. This article focuses on the common linear-by-linear interaction based on a moderated multiple regression model of the form

(1) $$\begin{array}{r} y_{i}=\beta_{0}+\beta_{1}T_{i}+\beta_{2}M_{i}+\beta_{3}\left(T_{i}\times M_{i}\right)+e_{i}, \end{array}$$

where *Y*, *T*, and *M* are an outcome, a categorical or continuous target predictor, and a continuous moderator variable, respectively, the βs are fixed unstandardized model parameters to be estimated, $e_{i}\sim N\left(0,\sigma^{2}\right)$ is a residual, and *i* indexes the individual cases in the data. In this model, the primary parameter of interest is β_3_ which, if non-zero, implies that the linear relationship between *Y* and *T* varies linearly with *M*. Several sources discuss inferential, computational, and interpretational approaches to moderated multiple regression (e.g., Aiken and West, 1991; Cohen et al., 2003; Bauer and Curran, 2005; Preacher et al., 2006; Hayes and Matthes, 2009; Aiken et al., 2012; Dawson, 2014; Hayes, 2013; Bodner, 2016).

After inferential evidence of an interactive effect (e.g., either using a null hypothesis significance test or confidence interval for β_3_), researchers turn to interpreting and communicating the nature of that effect, focusing on the conditional effects of the target predictor *T* on the outcome variable *Y* at specified values of the moderator variable *M*. Rearranging terms in Equation (1) such that

(2) $$\begin{array}{r} y_{i}=\left(\beta_{0}+\beta_{2}M_{i}\right)+\left(\beta_{1}+\beta_{3}M_{i}\right)T_{i}+e_{i}, \end{array}$$

the quantity β_1_ + β_3_*M*_*i*_ represents the conditional effect of *T* on *Y* for a given value of *M* and is sometimes referred to as the “simple slope” (e.g., Aiken and West, 1991). This article revisits how researchers might judge the practical magnitude of these conditional effects and differences in these conditional effects in light of not-so-recent calls for researchers to include information on the practical magnitude of effects (e.g., using effect sizes) to distinguish between the statistical and practical significance of research results (e.g., Wilkinson and Task Force on Statistical Inference, 1999).

When all three of the variables *Y*, *T*, and *M* have non-arbitrary and readily interpretable metrics (e.g., annual salary in dollars, an indicator of participant gender, and the number of years of education, respectively), the direction and magnitude of these conditional effects and differences in conditional effects are straightforward. Each conditional effect is an unstandardized effect size and researchers often present two or more of these conditional effects for the purpose of interpreting the nature of the statistical interaction in the text, tables, or figures of research reports. In these cases, there is a general consensus that unstandardized (i.e., simple) effect sizes are preferred over standardized effect sizes (for a recent review, see Baguley, 2009).

Standardized effect sizes are often recommended when at least one of the variables involved in the effect of interest has an arbitrary metric that is not readily interpretable in isolation (e.g., Kelley, 2007), which is often the case for variables in the social and behavior sciences (e.g., the occupational prestige scores, assessments of liking and assessments of the believed prevalence of sex discrimination in society that appear in the examples below). Although the use of standardized effect sizes to represent (unmoderated) associations is now widespread in many research literatures, their use to represent the conditional effects underlying statistical interactions, however, remains relatively rare despite several sources that describe and debate how this could be accomplished (e.g., Arnold, 1982; Stone and Hollenbeck, 1989). Instead, researchers most often focus on unstandardized conditional effects even in cases where one or more of the variables involved are in an arbitrary metric; in such cases, the practical magnitude of these conditional effects is uncertain. In order to improve research practices, this article demonstrates how unstandardized conditional effects can be transformed into standardized conditional effects for this purpose.

Before proceeding, I emphasize two cautionary notes raised in a recent article on moderated effect sizes (Smithson and Shou, 2016). First, there are numerous standardized effect sizes available to represent the conditional effects underlying statistical interactions (e.g., standardized partial regression coefficients, semi-partial correlation coefficients, partial correlation coefficients, conditional standardized mean differences) and none should be considered superior in all research contexts. This article focuses on the conditional standardized mean difference and the semi-partial correlation but any of the other alternatives may be useful. Second, the uniform transformation of unstandardized to standardized conditional effects requires several assumptions about the data (e.g., Arnold, 1982; Smithson, 2012; Smithson and Shou, 2016). These assumptions include, but are not limited to, a constant variance ratio $\sigma_{Y}^{2}/\sigma_{T}^{2}$ across all values of *M* and the absence of additional moderator effects involving *Y* in the same model. When either assumption does not hold, the direction and magnitude of standardized conditional effects can differ in important ways depending on the standardizing approach taken (Smithson and Shou, 2016). Smithson (2012) provides tests for the constant variance ratio assumption for categorical and continuous moderator variables but highlights the need for further methodological research for the continuous moderator variable case. Awaiting these developments, the approach discussed in this article assumes a constant variance ratio and the absence of other moderated effects.

This article is structured as follows. The following section focuses on the case where the target predictor *T* is a dichotomous indicator of group membership and demonstrates how the standardized mean difference can be adapted to provide information on the practical magnitude of moderated group mean differences. The subsequent section focuses on the case where the target predictor *T* is a continuous variable and demonstrates how the semi-partial correlation can be adapted to provide information on the practical magnitude of moderated linear associations. In both sections, the proposed approach is illustrated with a real data example and is shown to produce similar conclusions irrespective of whether researchers focus on the conditional effects at conventional values of the moderator variable *M* (e.g., at 1 *SD* below and above the moderator variable mean; Aiken and West, 1991; Cohen et al., 2003; Dawson, 2014) or across the across the range of moderator variable values (e.g., Hayes, 2013) supplemented with confidence interval information using the Johnson-Neyman (J-N) procedure (Johnson and Neyman, 1936; Potthoff, 1964; Rogosa, 1981; Bauer and Curran, 2005; Preacher et al., 2006; Hayes and Matthes, 2009). The concluding section provides further comments and extensions.

## 1. Moderated group mean differences

In this section, I develop and demonstrate the utility of a standardized effect size for group mean differences that varies linearly across the range of continuous moderator variable *M*-values. In situations without a moderator variable, the standardized mean difference (Cohen, 1988)

(3) $$\begin{array}{r} \delta_{Y}=\frac{\mu_{Y|A}-\mu_{Y|B}}{\sigma_{Y|g}} \end{array}$$

is a natural choice for a base-rate insensitive standardized effect size in contexts where the relative sample sizes in each group are not of substantive interest; in contexts where the relative sample sizes in each group are of substantive interest, the correlation-based approach in the next section might be more appropriate (McGrath and Meyer, 2006). In Equation (3), μ_*Y*|*A*_ and μ_*Y*|*B*_ are mean scores for variable *Y* in the populations that Group A and Group B represent, respectively, and σ_*Y*|*g*_ is the standard deviation of those scores which is assumed equal in each population. Ignoring the sign of δ_*Y*_, conventional guidelines for interpreting the magnitude of δ_*Y*_ are 0.2, 0.5, and 0.8, indicating small, medium, and large effects, respectively (Cohen, 1988). Equation (3) can be adapted easily to quantify the model-implied standardized mean difference at given values of moderator *M* to represent standardized conditional effects provided that the conditions identified in Smithson and Shou (2016) hold.

Consider the moderated multiple regression model in Equation (2) where *T* is a dichotomous indicator of group membership. Although any coding scheme for the dichotomous target predictor *T* is permissible (see e.g., Cohen et al., 2003, pp. 351–353), it is convenient to use a coding scheme that differs numerically by one unit (e.g., Group A = 1 and Group B = 0 or Group A = +0.5 and Group B = –0.5). Under this coding of the target predictor *T* and under the linearity assumptions of the model, the conditional effect β_1_ + β_3_*M*_*i*_ equals the model-implied difference in means on *Y* between the two groups at the specified of *M*. The variance of the residuals $e_{i}\sim N\left(0,\sigma^{2}\right)$ in this model represents random variability in *Y* within each group at a given value of *M* and is assumed homogeneous across groups and for all values of *M* under the model. Thus, under Smithson and Shou's (2016) identified conditions, the model-implied standardized mean difference at a given value of *M* is

(4) $$\begin{array}{r} \delta_{Y|M_{i}}=\frac{\beta_{1}+\beta_{3}M_{i}}{\sigma } \end{array}$$

which is estimated by

(5) $$\begin{array}{r} {\hat{\delta }}_{Y|M_{i}}=\frac{{\hat{\beta }}_{1}+{\hat{\beta }}_{3}M_{i}}{\sqrt{MSR}}, \end{array}$$

where *MSR* is the Mean Square Residual from the regression model's ANOVA summary table which is the pooled residual variance in *Y* across groups and is an unbiased estimator of σ^2^.

Given model estimates, a researcher can easily compute the implied group mean difference (i.e., ${\hat{\beta }}_{1}+{\hat{\beta }}_{3}M_{i}$) for each value of *M* in the data and then divide these values by $\sqrt{MSR}$ as in Equation (5) to yield a linear progression of ${\hat{\delta }}_{Y|M_{i}}$ values across the observed values of *M*. These ${\hat{\delta }}_{Y|M_{i}}$ values can be interpreted in isolation (i.e., the standardized mean difference at a given value of *M*) and differences in ${\hat{\delta }}_{Y|M_{i}}$-values can be discussed (e.g., the range of standardized mean differences across a range of *M* values). Importantly, the directions and magnitudes of these standardized effects can be evaluated using conventional guidelines for standardized mean differences. Although guidelines do not exist for the comparison of standardized mean differences across a range of moderator variable values, I propose that $\Delta {\hat{\delta }}_{Y|M_{i}}=$ 0.4, 1.0, and 1.6 could be considered “small,” “medium,” and “large” differences, respectively, for a 2 standard deviation difference in the moderator variable *M* (e.g., from 1 *SD* below to 1 *SD* above the moderator variable mean), a moderator variable range often used when interpreting interactions^1^. These proposed criterion values yield similar qualitative conclusions about the magnitude of an interactive effect based on Green's (1991) revision of Cohen's (1988) guidelines for an effect in multiple regression as illustrated in the following example^2^.

### 1.1. Example

The data for this example are drawn from a published experimental study (Garcia et al., 2010) and used as an example in Hayes (2013)^3^. Participants read a vignette about a female attorney who lost a promotion due to sex discrimination. Participants were randomly assigned to experimental conditions where the attorney either protested or did not protest the promotion decision. After reading the vignette, participants evaluated the female attorney on six dimensions which were averaged to represent an overall measure of liking. Furthermore, participants completed an eight-item measure assessing their beliefs about the pervasiveness of sexual discrimination in society (PSD) and item responses were averaged. Of interest was whether and how the effect of the protest manipulation on liking varied across levels of PSD. Note that the liking and PSD variable scores do not have natural and readily understandable metrics. Table 1 provides descriptive statistics for these variables, noting that the correlation between liking and the protest condition indicator is positive and statistically significant. In the standardized mean difference metric, the unmoderated effect of the protest condition on liking would be considered “medium to large” in size (i.e., ${\hat{\delta }}_{Y}=0.46$).

**Table 1:** **Descriptive statistics for protest condition, liking, and pervasiveness of sex discrimination**.

					**Correlations**		
**Variable**	**Min**.	**Max**.	***M***	***SD***	**1**	**2**	**3**
1. Liking	1.00	7.00	5.64	1.05	1.00	.	.
2. Protest condition	0	1	0.68	0.47	0.21^*^	1.00	.
3. Pervasiveness of sex discrimination	2.87	7.00	5.12	0.78	0.09	0.04	1.00

*N = 129*.

* *p < 0.05. Protest condition coding: No Protest = 0, Protest = 1*.

Table 2 provides the moderated multiple regression analysis results. The significant interactive effect of the protest condition and PSD on liking, ${\hat{\beta }}_{3}$ = 0.83, *t*_(125)_ = 3.42, *p* = 0.001, indicates that the effect of protest condition on liking varies linearly with PSD; the magnitude of this interactive effect might be considered “medium” (i.e., Δ*R*^2^ = 0.085; Green, 1991). Below, the two standard approaches are used to investigate the nature of this interaction, graphs of conditional effects at select moderator variable values and graphs of conditional effects across the observed range of the moderator variable values. In each approach, the standardizing transformation for conditional effects in Equation (5) is used and the equivalence of results irrespective of approach is demonstrated when the recommendations are followed.

**Table 2:** **Model parameters from the regression of liking on protest condition, believed pervasiveness of sex discrimination, and their interaction**.

	**Estimate**	***SE***	***t*_(125)_**	***p***
Intercept	7.71	1.05	7.38	<0.001
Protest condition (PROTEST)	–3.77	1.25	–3.01	0.003
Pervasiveness of sex discrimination (PSD)	–0.47	0.20	–2.32	0.022
PROTEST × PSD	0.83	0.24	3.42	0.001

*N = 129. Model R^2^ = 0.13, F_(3, 125)_ = 6.42, p < 0.001, MSR = 0.978. Protest condition coding: No Protest = 0, Protest = 1*.

#### 1.1.1. Conditional effects at select moderator values

**Figure 1:**
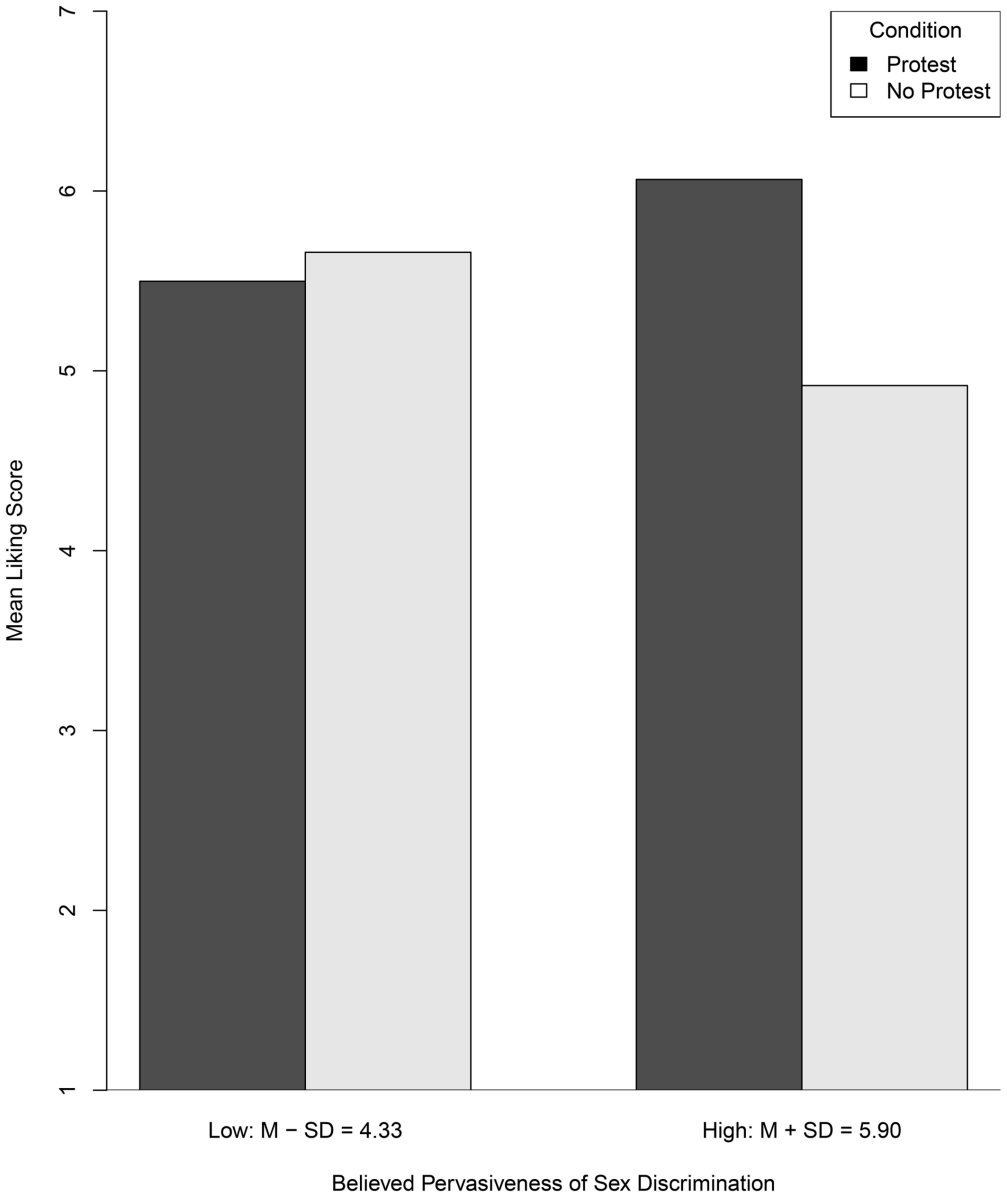
**Model-based mean liking scores as a function of protest condition evaluated at 1 SD above and below the mean of Pervasiveness of Sex Discrimination**.

Figure 1 provides a graph depicting the nature of this interactive effect where the conditional effect of the protest manipulation on liking (i.e., the difference in heights of adjacent bars) is evaluated at 1 *SD* below and above the mean of PSD. Descriptively, at one standard deviation above the PSD mean, participants liked the female attorney more on average in the protest condition than in the no-protest condition (i.e., ${\hat{\beta }}_{1}+{\hat{\beta }}_{3}M_{i}=-3.773\;+\;0.834\times 5.901=1.148$); at one standard deviation below the PSD mean, this effect was smaller and opposite in direction (i.e., ${\hat{\beta }}_{1}+{\hat{\beta }}_{3}M_{i}=-3.773+0.834\times 4.333=-0.159$). The practical magnitude of these two conditional effects and the difference in these two conditional effects, however, is not clear. In the standardized mean difference metric applying Equation (5), these standardized conditional effects are ${\hat{\delta }}_{Y|M_{i}}=1.148/\sqrt{0.978}=1.160$ and ${\hat{\delta }}_{Y|M_{i}}=-0.159/\sqrt{0.978}=-0.161$, respectively. Using conventional guidelines, the former would be considered positive and “large” and the latter would be considered negative and “small.” Using the proposed guidelines, the difference in these values [i.e., 1.160 – (–0.161) = 1.321] for a 2 *SD* increase in PSD would be considered “medium” in magnitude.

#### 1.1.2. Conditional effects across a range of moderator values

**Figure 2:**
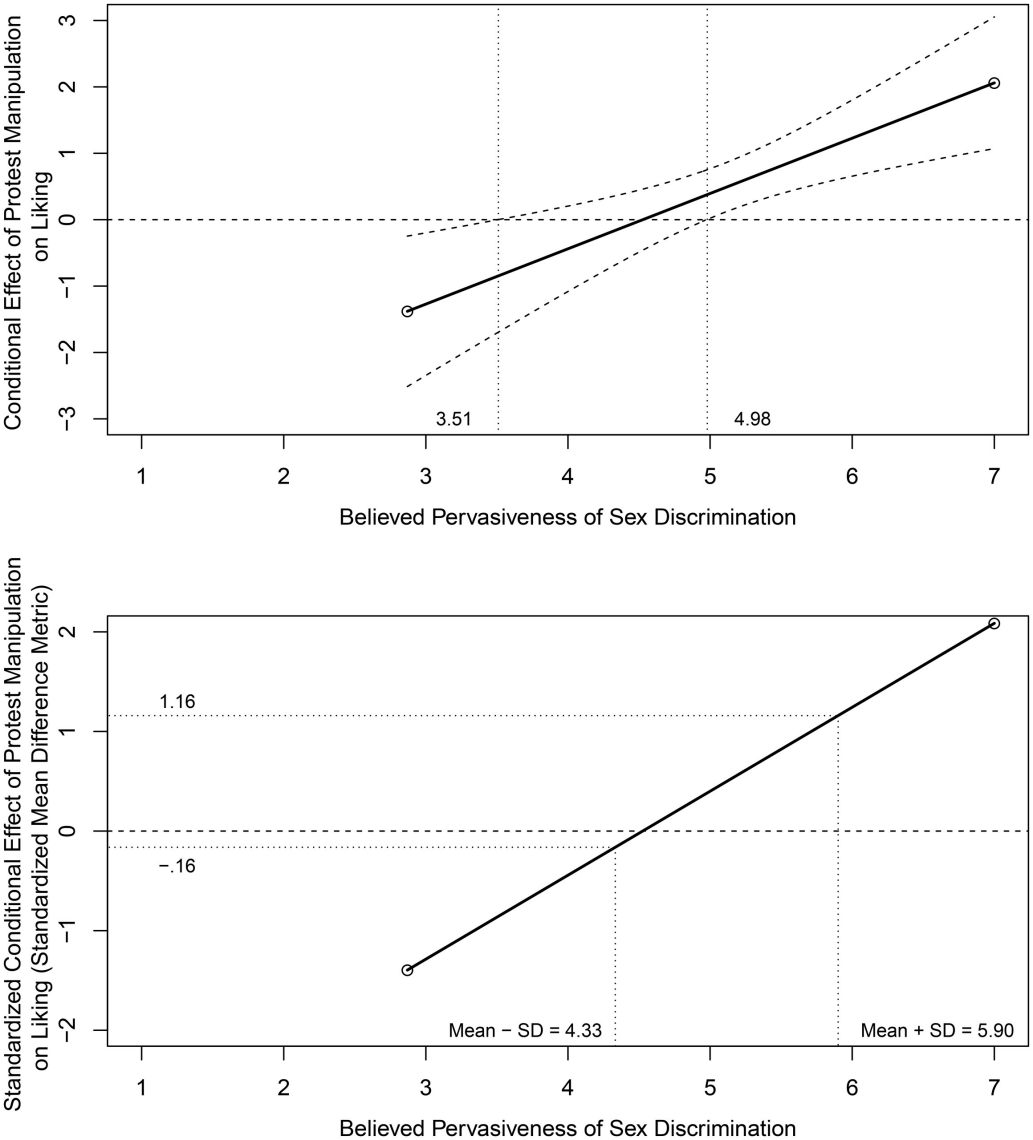
**Conditional effect with Johnson-Neyman confidence limits (top panel) and standardized conditional effect (bottom panel) of protest condition on liking as a function of Pervasiveness of Sex Discrimination**. Top panel modified from Figure 7.8 in Hayes (2013) and reprinted with permission of Guilford Press.

The top panel of Figure 2 provides a plot of the conditional effect of protest condition on liking as a function of PSD based on output from the PROCESS software program with the Johnson-Neyman (J-N) option (Hayes, 2013); the slope of the conditional effects line equals the interaction parameter value in Table 2. The J-N analysis indicates that the attorney's protest behavior had a significantly positive effect on liking for participants with a PSD score above 4.98 and a significantly negative effect on liking for participants with a PSD score <3.51. Hayes (2013, pp. 243–244) notes that only two participants have scores lower than this latter criterion value and should therefore be interpreted with caution. The practical magnitude of these conditional effects and differences in conditional effects across the range of PSD, however, is not clear.

The bottom panel of Figure 2 provides a *standardized* conditional effects display in the standardized mean difference metric applying the standardizing transformation in Equation (5). These standardized mean differences range from ${\hat{\delta }}_{Y|M_{i}}=\left(-3.773+0.834\times 2.870\right)/\sqrt{0.978}=-1.395$ at the minimum observed PSD value to ${\hat{\delta }}_{Y|M_{i}}=\left(-3.773+0.834\times 7.000\right)/\sqrt{0.978}=2.088$ at the maximum observed PSD value. Using conventional guidelines, these standardized mean differences range from negative and “large” to positive and “large” across the observed range of PSD. To minimize the effects of sparse tails or extreme values in the distribution of PSD, primary attention should focus on the range of standardized mean differences from 1 *SD* below the mean to 1 *SD* above the mean of PSD which is typical when graphing and interpreting interactive effects. These standardized mean differences range from ${\hat{\delta }}_{Y|M_{i}}=\left(-3.773+0.834\times 4.333\right)/\sqrt{0.978}=-0.161$ to ${\hat{\delta }}_{Y|M_{i}}=\left(-3.773+0.834\times 5.901\right)/\sqrt{0.978}=1.160$ and individually would be considered negative and “small” and positive and “large,” respectively, using conventional guidelines. Using the proposed guidelines, the difference in these boundary values [i.e., 1.160 – (–0.161) = 1.321] for a 2 *SD* increase in PSD would be considered “medium” in magnitude.

## 2. Moderated linear associations

In this section, I develop and demonstrate the utility of a standardized effect size for linear associations that varies linearly across the range of continuous moderator variable *M*-values. In situations without a moderator variable, the correlation coefficient

(6) $$\begin{array}{r} \rho_{Y,T}=\frac{Cov\left[Y,T\right]}{SD\left[Y\right]SD\left[T\right]} \end{array}$$

is a natural choice for a standardized effect size (Cohen, 1988). In Equation (6), *Cov*[*Y, T*] is the covariance between variables *Y* and *T* with standard deviations *SD*[*Y*] and *SD*[*T*], respectively, in the population of interest. Ignoring the sign of ρ_*Y, T*_, conventional guidelines for interpreting the magnitude of ρ_*Y, T*_ are 0.1, 0.3, and 0.5, indicating small, medium, and large effects, respectively (Cohen, 1988). Linear associations in a multiple regression model, however, statistically control for the relationship between the target predictor variable *T* and the moderator variable *M*. Thus, one measure of this linear association is the semi-partial correlation coefficient,

(7) $$\begin{array}{r} \rho_{Y,\left(T|M_{i}\right)}=\frac{Cov\left[Y,T|M_{i}\right]}{SD\left[Y\right]SD\left[T|M_{i}\right]}, \end{array}$$

where the variable *T*|*M*_*i*_ (read *T* given *M*_*i*_) is the residual of the variable *T* after accounting for its linear relationship with *M* at *M*_*i*_ (e.g., Pedhazur, 1997; Maxwell, 2000; Cohen et al., 2003). Below, the conventional guidelines for ρ_*Y, T*_ are used for interpreting the practical magnitude of ρ_*Y*, (*T*|_*M*__*i*_)_ (see Maxwell, 2000, pp. 438–439, for a more complete discussion of alternative guidelines that differ only slightly). Equation (7) can easily be adapted to quantify the model-implied semi-partial correlation between outcome variable *Y* and target predictor *T* at given values of moderator *M* provided that the conditions identified in Smithson and Shou (2016) hold.

Consider the moderated multiple regression model in Equation (2) where *T* is a continuous variable; the conditional effect β_1_ + β_3_*M*_*i*_ equals the model-implied unstandardized linear regression coefficient for outcome variable *Y* from target predictor *T* at the specified value of *M*. Under the linearity assumptions of the model, these conditional effects are equal to

(8) $$\begin{array}{r} \beta_{1}+\beta_{3}M_{i}=\frac{Cov\left(Y,T|M_{i}\right)}{Var\left(T|M_{i}\right)}. \end{array}$$

Combining Equations (7, 8) and under and Smithson and Shou's (2016) identified conditions, the model-implied semi-partial correlation between *Y* and *T* for a given value of *M* equals

(9) $$\begin{array}{r} \rho_{Y,\left(T|M_{i}\right)}=\left(\beta_{1}+\beta_{3}M_{i}\right)\frac{SD\left(T|M_{i}\right)}{SD\left(Y\right)}. \end{array}$$

If it is reasonable to assume that the residuals in the regression of *T* on *M* are homoscedastic (cf. Smithson and Shou, 2016), then *SD*(*T*|*M*_*i*_) = *SD*(*T*|*M*) and

(10) $$\begin{array}{r} \rho_{Y,\left(T|M_{i}\right)}=\left(\beta_{1}+\beta_{3}M_{i}\right)\frac{SD\left(T|M\right)}{SD\left(Y\right)} \end{array}$$

which is estimated by

(11) $$\begin{array}{r} {\hat{\rho }}_{Y,\left(T|M_{i}\right)}=\left({\hat{\beta }}_{1}+{\hat{\beta }}_{3}M_{i}\right)\frac{\hat{SD}\left(T|M\right)}{\hat{SD}\left(Y\right)}. \end{array}$$

Note that *SD*(*T*|*M*) is estimated by the square root of the Mean Square Residual (MSR) from the ANOVA summary table in the regression of *T* on *M*.

Given model estimates, a researcher can easily compute the model-implied unstandardized regression weight for the linear relationship between *Y* and *T* for each value of *M* in the data (i.e., ${\hat{\beta }}_{1}+{\hat{\beta }}_{3}M_{i}$) and then multiply these estimates by the ratio of $\hat{SD}\left(T|M\right)/\hat{SD}\left(Y\right)$ as in Equation (11) to yield a linear progression of ${\hat{\rho }}_{Y,\left(T|M_{i}\right)}$ values across the observed values of *M*. These ${\hat{\rho }}_{Y,\left(T|M_{i}\right)}$ values can be interpreted in isolation (i.e., the semi-partial correlation between *Y* and *T* at a given value of *M*) and differences in ${\hat{\rho }}_{Y,\left(T|M_{i}\right)}$ values can be discussed (e.g., the range of semi-partial correlations between *Y* and *T* across a range of *M* values). Importantly, the directions and magnitudes of these standardized conditional effects can be evaluated using conventional guidelines for semi-partial correlations. Although guidelines do not exist for the comparison of semi-partial correlations across a range of moderator variable values, I propose that $\Delta {\hat{\rho }}_{Y,\left(T|M_{i}\right)}=$ 0.14, 0.42, and 0.71 could be considered “small,” “medium,” and “large” differences, respectively, for a two standard deviation difference in the moderator variable *M* (e.g., from 1 *SD* below to 1 *SD* above the moderator variable mean), a moderator variable range often used when interpreting interactions^4^. These proposed criterion values yield similar qualitative conclusions about the magnitude of an interactive effect based on Green's (1991) revision of Cohen's (1988) guidelines for an effect in multiple regression as illustrated in the following example.

### 2.1. Example

The data for this example are taken from the 1991 US General Social Survey (Smith et al., 2011) and focuses on how the relationship between a respondent's years of education (EDUC) and occupational prestige varies as a function of the respondent's mother's years of education (MEDUC)^5^. Note that the occupational prestige scores do not have a natural and readily understandable metric. Table 3 provides the descriptive statistics for these three variables, noting that the correlation between occupational prestige and EDUC (*r* = 0.51) is positive, large in size, and statistically significant.

**Table 3:** **Descriptive statistics for respondent's occupational prestige, years of education, and mother's years of education**.

					**Correlations**		
**Variable**	**Min**.	**Max**.	***M***	***SD***	**1**	**2**	**3**
1. Occupational prestige	17	86	43.67	13.26	1.00	.	.
2. Years of education	0	20	13.41	2.80	0.51^*^	1.00	.
3. Mother's years of education	0	20	10.79	3.44	0.15^*^	0.42^*^	1.00

*Notes: N = 1,162*.

* *p < 0.05*.

Table 4 provides the moderated multiple regression analysis results. The significant interactive effect of EDUC and MEDUC on occupational prestige, ${\hat{\beta }}_{3}=0.08,t_{\left(1158\right)}=2.97,p=0.003$, indicates that the linear relationship between a respondent's occupational prestige and years of education varies linearly with the respondent's mother's years of education; the magnitude of this interactive effect might be considered “small” (i.e., Δ*R*^2^ = 0.008; see Footnote 2). As in the prior example, the two standard approaches are used to investigate the nature of this interaction, graphs of conditional effects at select moderator variable values and graphs of conditional effects across the observed range of the moderator variable values. In each approach, the standardizing transformation for conditional effects in Equation (11) is used and the equivalence of results irrespective of approach is demonstrated when the recommendations are followed.

**Table 4:** **Model parameters from the regression of occupational prestige on years of education, mother's years of education, and their interaction**.

	**Estimate**	***SE***	***t*_(1158)_**	**p**
Intercept	22.83	3.85	5.93	<0.001
Years of education (EDUC)	1.75	0.30	5.79	<0.001
Mother's years of education (MEDUC)	–1.36	0.38	–3.63	<0.001
EDUC × MEDUC	0.08	0.03	2.97	0.003

*N = 1,162. Model R^2^ = 0.27, F_(3, 1158)_=141.25, p < 0.001*.

#### 2.1.1. Conditional effects at select moderator values

**Figure 3:**
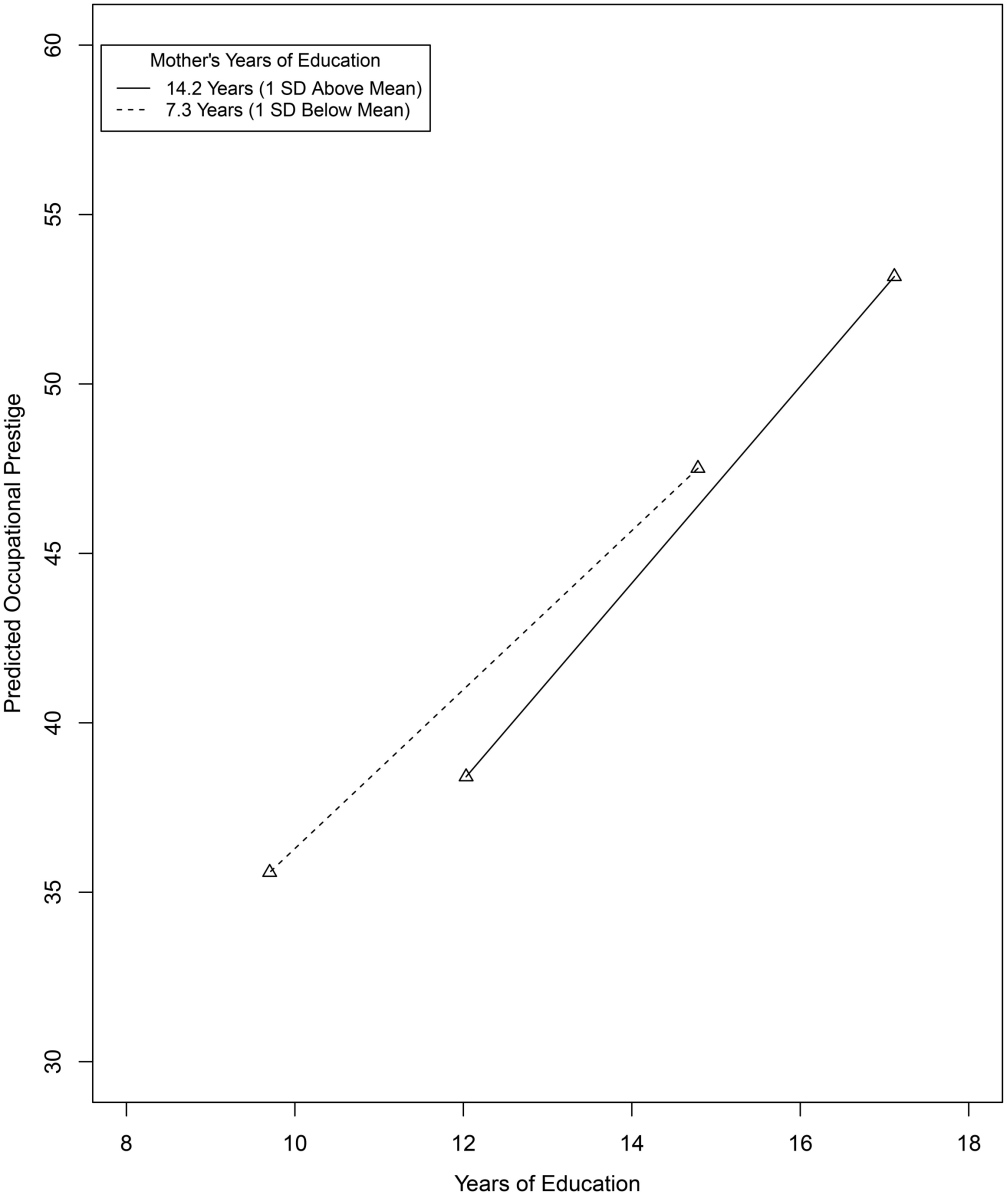
**Tumble graph of the relationship between respondent's years of education and occupational prestige evaluated at 1 SD above and below the mean of respondent's mother's years of education**.

Figure 3 provides a Tumble graph (Bodner, 2016) depicting the nature of this interactive effect where the conditional effect of respondent's education on occupational prestige (i.e., the slopes of the plotted line segments) is evaluated at 1 *SD* below and above the mean of MEDUC^6^. Descriptively, at one standard deviation below the MEDUC mean, the linear relationship between a respondent's years of education and occupational prestige is positive (${\hat{\beta }}_{1}+{\hat{\beta }}_{3}M_{i}=1.749+0.081\times 7.347=2.345$); at one standard deviation above the MEDUC mean, this linear relationship is more strongly positive (${\hat{\beta }}_{1}+{\hat{\beta }}_{3}M_{i}=1.749+0.081\times 14.233=2.902$). The practical magnitude of these two conditional effects and the difference in these two conditional effects, however, is not clear. In the semi-partial correlation metric applying the transformation in Equation (11), these standardized conditional effects are ${\hat{\rho }}_{Y,\left(T|M_{i}\right)}=2.345\times \left(\sqrt{6.462}/13.255\right)=0.45$ and ${\hat{\rho }}_{Y,\left(T|M_{i}\right)}=2.902\times \left(\sqrt{6.462}/13.255\right)=0.56$, respectively^7^. Using conventional guidelines, these semi-partial correlations individually would be considered positive and “medium to large” and “large,” respectively. Using the proposed guidelines, the difference in these values (i.e., 0.56 – 0.45 = 0.11) for a 2 *SD* increase in mother's years of education would be considered “small” in magnitude.

#### 2.1.2. Conditional effects across a range of moderator values

**Figure 4:**
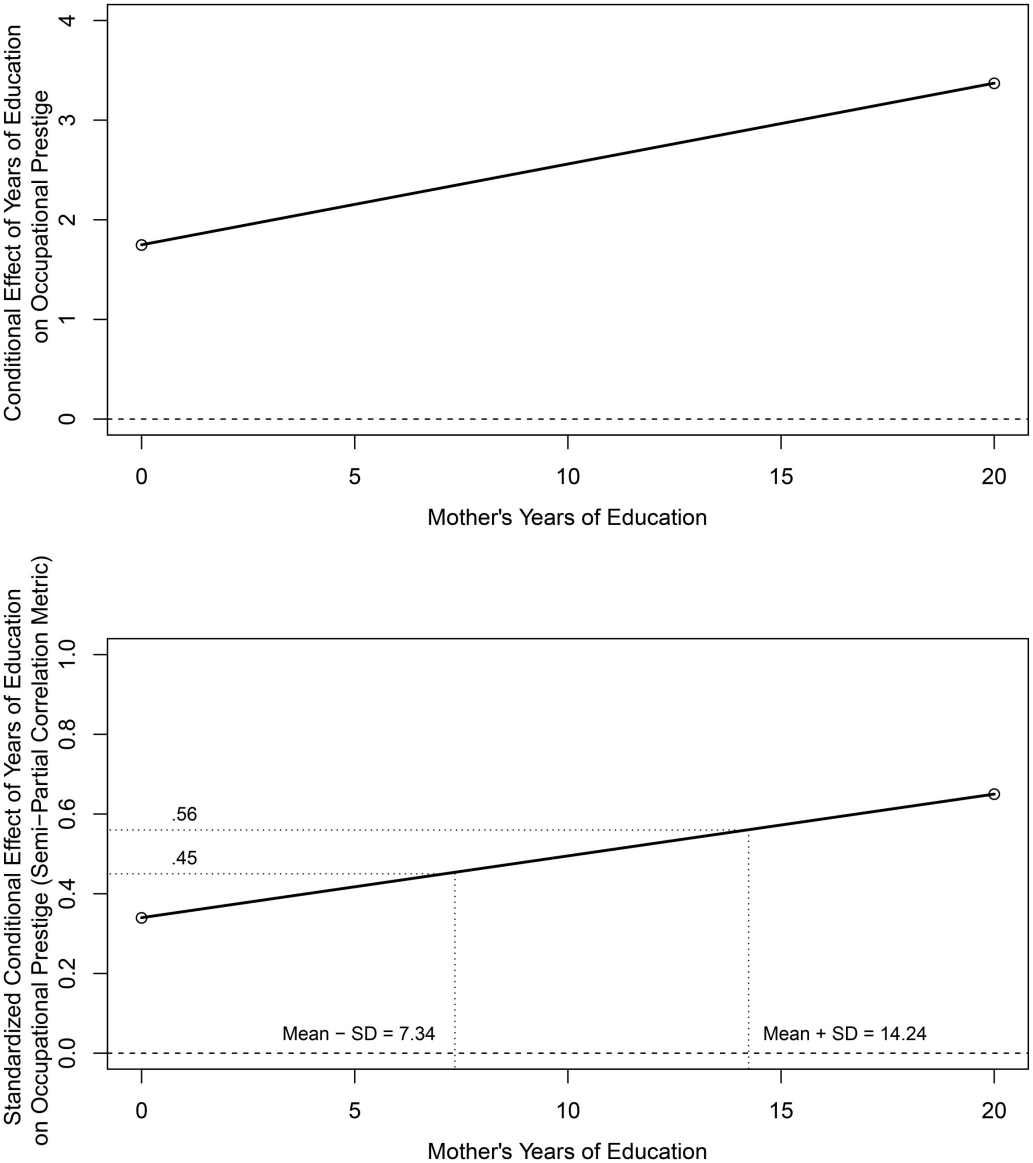
**Conditional effect (top panel) and standardized conditional effect (bottom panel) of respondent's years of education on occupational prestige as a function of respondent's mother's years of education**.

The top panel of Figure 4 provides a plot of the conditional effect of years of education on occupation prestige as a function of the years of mother's education based on output from the PROCESS software program using the Johnson-Neyman (J-N) option (Hayes, 2013); the slope of the conditional effects line equals the interaction parameter value in Table 4. The analysis indicated that the these conditional effects are positive and significant for all observed values of mother's education (i.e., there are no transitions in the regions of significance and therefore PROCESS does not provide confidence interval information). The practical magnitude of these conditional effects and differences in conditional effects across the range of mother's years of education, however, is not clear.

The bottom panel of Figure 4 provides a *standardized* conditional effects display using the semi-partial correlation metric applying the standardizing transformation in Equation (11). These semi-partial correlations range from ${\hat{\rho }}_{Y,\left(T|M_{i}\right)}=\left(1.749+0.081\times 0\right)\left(\sqrt{6.462}/13.255\right)=0.34$ for a mother with 0 years of education to ${\hat{\rho }}_{Y,\left(T|M_{i}\right)}=\left(1.749+0.081\times 20\right)\left(\sqrt{6.462}/13.255\right)=0.65$ for a mother with 20 years of education, the minimum and maximum observed values in the data set, respectively. Using the guidelines for interpreting the magnitude of correlations, these correlations range from positive and “medium” to positive and “large” across the range of years of mother's education. To minimize the effects of sparse tails or extreme values in the distribution of mother's years of education, primary attention should focus on the range of semi-partial correlations from 1 SD below the mean (i.e., 7.347 years) to 1 *SD* above the mean (i.e., 14.233 years) of mother's education which is typical when graphing and interpreting interactive effects. These semi-partial correlations range from ${\hat{\rho }}_{Y,\left(T|M_{i}\right)}=\left(1.749+0.081\times 7.347\right)\left(\sqrt{6.462}/13.255\right)=0.45$ to ${\hat{\rho }}_{Y,\left(T|M_{i}\right)}=\left(1.749+0.081\times 14.233\right)\left(\sqrt{6.462}/13.255\right)=0.56$ and individually would be considered positive and “medium to large” and “large,” respectively. Using the proposed guidelines, the difference in these boundary values (i.e., 0.56 – 0.45 = 0.11) for a 2 *SD* increase in mother's years of education would be considered “small” in magnitude.

## 3. Concluding remarks

Wilkinson and Task Force on Statistical Inference (1999) recommended that researchers include information on the practical magnitude of effects (e.g., using effect sizes) to distinguish between the statistical and practical significance of research results. To date, however, this recommendation has not been widely incorporated into the interpretation and communication of the conditional effects and differences in conditional effects underlying statistical interactions when one or more of the variables involved in the interaction is in an arbitrary metric where standardized effect sizes are often useful. This article developed and presented a descriptive approach to investigate statistical interactions involving continuous moderator variables where the conditional effects underlying interactions are expressed in standardized effect size metrics. The proposed approach permits researchers to evaluate and communicate the practical magnitude of conditional effects and differences in conditional effects using conventional and proposed guidelines for the standardized effect size and therefore provides important supplementary information lacking under the two approaches researchers currently use that involve *unstandardized* conditional effects. The two real data examples illustrated the proposed approach and demonstrated its utility irrespective of whether a researcher chooses to interpret differences in conditional effects at specified moderator values or across the range of moderator variable values.

As noted in the introduction, other standardized effect size metrics than those presented are available; furthermore, the uniform translation of unstandardized to standardized conditional effects depends on several important assumptions about the data (Smithson and Shou, 2016). Although developing approaches to assess these assumptions for the case of continuous moderator variables is an active area of research (Smithson, 2012), researchers should routinely explore their plausibility. For example, researchers could use graphical approaches to assess visually whether the data contradict the assumption that the residual variances in *Y* and *T* are reasonably constant across the values of *M* (see e.g., Cohen et al., 2003, Ch. 4). In models with additional predictors not involved in the moderated effect of interest, additional moderated effects could be modeled and tested. Furthermore, the approach in this article also assumes that the model parameters of interest are fixed effects (i.e., the parameters of interest do not have random effects as appear in multilevel models); standardized effect sizes in random effects models critically depend on whether and how the random effect variance is used in the standardization process (Feingold, 2009). If the data appear to contradict these assumptions, a prudent course of action would be to focus on the unstandardized rather than the standardized conditional effects underlying the statistical interaction of interest; in such cases, judgements about the practical magnitude of the unstandardized conditional effects, however, will be uncertain.

The approach described in this article is invariant to whether or not the moderator variable is grand-mean centered. Consider that only the value of β_1_ in Equations (4, 10) might change depending on whether or not the moderator variable is grand-mean centered; all other quantities, other than the user-specified values for *M*_*i*_, are invariant. To ensure accurate results, the value of β_1_ used when computing conditional effects and standardized conditional effects must match the moderator variable values used to estimate the model parameters (i.e., mean centered or not mean centered). To illustrate, reconsider the example where the effect of the protest condition on liking depended on PSD. This example analysis did not grand-mean center PSD and yielded a standardized conditional effect of the protest condition on liking at 1 *SD* above the PSD mean of ${\hat{\delta }}_{Y|M_{i}}=\left(-3.773+0.834\times 5.901\right)/\sqrt{0.978}=1.160$. When PSD is grand-mean centered and the analysis is re-conducted with these values, the same standardized conditional effect is obtained, i.e., ${\hat{\delta }}_{Y|M_{i}}\;=\;\left(0.493+0.834\times 0.784\right)/\sqrt{0.978}=1.160$; here ${\hat{\beta }}_{1}=0.493$ and *M*_*i*_ = 0.784, the latter reflecting a moderator variable value 1 *SD* above the mean centered PSD mean of zero. An erroneous quantity would result if the non-centered moderator value at 1 *SD* above the raw PSD mean was used with the ${\hat{\beta }}_{1}$ from the model based on mean-centered PSD values, i.e., ${\hat{\delta }}_{Y|M_{i}}=\left(0.493+0.834\times 5.901\right)/\sqrt{0.978}=5.475$.

The proposed guidelines for interpreting the magnitude of a difference in standardized conditional effects for 2 *SD* difference in the continuous moderator variable should be considered provisional and future research is needed to verify or recommend adjustments to these guidelines. Although the proposed guidelines provide similar qualitative conclusions for the practical magnitude of an interactive effect based on Green (1991) revision of Cohen (1988) guidelines for partial effects in a multiple regression context, as illustrated in the two examples, researchers have noted that the magnitude of interactive effects in the social and behavioral sciences tend to be even smaller than these criterion values (e.g., McClelland and Judd, 1993; Aguinis et al., 2005). Revisions to the proposed magnitude guidelines could be based on empirical distributions for the difference in standardized conditional effects for a 2 *SD* difference in the moderator variable gleaned from research reports. I encourage study authors to provide these values in research reports to facilitate this research.

Future research may also explore extensions of the J-N approach to place confidence intervals around standardized conditional effects and differences in standardized conditional effects. In doing so, care should be taken as confidence interval construction around standardized effects requires a more sophisticated approach than for unstandardized effect sizes (cf. Cumming and Finch, 2001; Smithson, 2001; Kelley, 2007). Although developing such procedures would be a nice technical contribution, this approach is not considered here as the focus is on descriptive aspects of the assumed smooth linear regression surface in Equation (1) (i.e., without points of discontinuity that regions of significance imply). The regions of significance approach underlying the J-N procedure, in contrast, is an inferential procedure influenced by sample size (cf. Potthoff, 1964) that inadvertently may encourage researchers to conclude erroneously that non-significant conditional effects (standardized or unstandardized) are equal to zero instead of following a smooth linear function through significant and non-significant regions as in the top panel of Figure 2 (cf. Dawson, 2014).

## Author contributions

As sole author, TB contributed to all aspects of the research in the submitted manuscript.

## Funding

This article's publication was funded by the Portland State University Open Access Article Processing Charge Fund, administered by the Portland State University Library.

### Conflict of interest statement

The author declares that the research was conducted in the absence of any commercial or financial relationships that could be construed as a potential conflict of interest.

## Footnotes

^1^Computed as $2\times \left({\hat{\delta }}_{Y|M_{i}}=0.2,0.5,\;\mathrm{and}\;.8\right)$.

^2^Green (1991) only redefines Cohen (1988) guideline for a medium effect to Δ*R*^2^ = 0.07, arguing that Cohen's guidelines for partial effects in a multiple regression context are too large relative to the effects observed in the social and behavioral sciences. Green's approach to redefine a medium partial effect can be used to provide revised guidelines for small and large partial effects. Consider the ratio of Green's definition of medium squared partial effect, which is equal to the squared semi-partial correlation for that effect, to Cohen's definition of medium squared zero-order correlation, (Δ*R*^2^ = 0.07)/(ρ^2^ = 0.3^2^ = 0.09) = 0.778. Using this constant of proportionality, Δ*R*^2^ = 0.008 and Δ*R*^2^ = 0.194 would be considered “small” and “large” partial effects, respectively.

^3^The data for this example are available for download at http://afhayes.com/introduction-to-mediation-moderation-and-conditional-process-analysis.html.

^4^These values are computed as $\sqrt{2\times \left({\hat{\rho }}_{Y,\left(T|M_{i}\right)}^{2}=0.1^{2},0.3^{2},\;\mathrm{and}\;0.5^{2}\right)}$ to account for the fact that the absolute value of each ${\hat{\rho }}_{Y,\left(T|M_{i}\right)}$ must be ≤ 1.00.

^5^I thank Dr. Dale Berger of Claremont Graduate University for this example. The data are downloadable at http://gss.norc.org/get-the-data/spss.

^6^Tumble graphs adjust the end-points of the plotted line segments to minimize the likelihood of plotting points in sparse or empty data regions when the target predictor and moderator variables are correlated.

^7^The MSR from the regression of EDUC on MEDUC is 6.462.
